# Successful treatment of ovarian cancer with apatinib combined with chemotherapy: A case report

**DOI:** 10.1097/MD.0000000000008570

**Published:** 2017-11-10

**Authors:** Mingzi Zhang, Zhongkai Tian, Yehong Sun

**Affiliations:** a Department of Pharmacy, Shenzhen Traditional Chinese Medicine Hospital; b Department of Internal Medicine-Oncology, Second Affiliated Hospital of Jinan University, Shenzhen People's Hospital, Shenzhen, Guangdong, People's Republic of China.

**Keywords:** adenocarcinoma, angiogenesis inhibitor, apatinib, maintenance therapy, ovarian cancer

## Abstract

**Rationale:**

The standard treatment for ovarian cancer is chemotherapy with 2 drugs (taxanes and platinum drugs). However, the traditional combination of the 2 drugs has many adverse effects (AEs) and the cancer cells will quickly become resistant to the drugs. Apatinib is a small-molecule antiangiogenic agent which has shown promising therapeutic effects against diverse tumor types, but it still remains unknown whether apatinib has an antitumor effect in patients with ovarian cancer. Herein, we present a successfully treated case of ovarian cancer using chemotherapy and apatinib, in order to demonstrate the effectiveness of this new combined regimen in ovarian cancer.

**Patients concerns:**

A 51-year-old Chinese woman presented with ovarian cancer >4.5 years. The disease and the cancer antigen 125 (CA-125) had been controlled well by surgical treatment and following chemotherapy. However, the drugs could not control the disease anymore as the CA-125 level was significantly increasing.

**Diagnosis:**

Ovarian cancer.

**Interventions:**

The patient was treated with apatinib combined with epirubicin. Apatinib was administered orally, at an initial daily dose of 500 mg, and was then reduced to 250 mg qd after the appearance of intolerable hand–foot syndrome (HFS) and oral ulcer. Then, the oral ulcer disappeared and the HFS was controlled by dose adjustment, oral vitamin B6, and hand cream application.

**Outcomes:**

The CA-125 reverted to the normal value after treatment with the new regimen. Magnetic resonance imaging showed that the original tumor lesions had disappeared. Apatinib monotherapy as maintenance therapy was then used to successfully control the cancer with a complete response. Our study is the first, to our knowledge, to report the therapeutic effects of apatinib and epirubicin on ovarian cancer.

**Lessons:**

Apatinib combined with chemotherapy and apatinib monotherapy as maintenance therapy could be a new therapeutic strategy for ovarian cancer, especially adenocarcinomas.

## 1 Introduction

Ovarian cancer is a malignant tumor posing a serious threat to global women's health. Approximately 70% of patients suffered ovarian cancer are diagnosed at a late stage of disease. Most of these tumors easily develop drug resistance during chemotherapy.^[1]^ Multidrug resistance (MDR) is the main cause of chemotherapy failure in ovarian cancer.^[2]^ Therefore, ovarian cancer has a poor prognosis, with only 40% of patients surviving for 5 years.^[3]^

Novel agents are being regularly developed to target specific molecular pathways. Numerous protein tyrosine kinase inhibitors (TKIs) and angiogenesis inhibitorswere found to be effective for the treatment of tumors including ovarian cancer when given alone or in combination with conventional chemotherapeutics, and the involved molecular mechanisms have been reported.^[4–6]^

Apatinib is a small-molecule antiangiogenic agent that selectively inhibits vascular endothelial growth factor receptor 2 (VEGFR-2) and also mildly inhibits c-Kit and c-Src tyrosine kinases.^[7]^ Angiogenesis is an essential step in tumor growth and metastasis. As is well known, VEGF signaling plays an important role in the angiogenic process of solid tumors. By binding to VEGFR-2, apatinib inhibits the effects of VEGF binding and subsequent VEGFR-2 autophosphorylation.^[8]^ Although apatinib has shown promising therapeutic effects against diverse tumor types in several phase II clinical trials, it still remains unknown whether apatinib has an antitumor effect in patients with ovarian cancer.^[8,9]^

Apatinib was approved and launched in the People's Republic of China in 2014 as a 3rd-line treatment for patients with advanced gastric or gastro-esophageal adenocarcinoma. A recent published phase-III trial showed that apatinib treatment significantly improved overall and progression-free survival with an acceptable safety profile in patients with advanced gastric cancer refractory to 2 or more lines of prior chemotherapy.^[10]^ It is also currently in phase II/III clinical trials in China for the treatment of many cancers, such as nonsmall-cell lung cancer, breast cancer, and hepatocellular carcinoma.^[9–12]^ Recently, apatinib is also reported having significant antitumor effects on tosarcoma such as osteosarcoma, pleomorphic liposarcoma, and angiosarcoma.^[13–15]^ These clinical trials and case reports demonstrated that apatinib has potential antitumor activity for various types of advanced solid tumors.

Herein, we present a successfully treated case of ovarian cancer using chemotherapy and apatinib, in order to demonstrate the effectiveness of this new combined regimen in ovarian cancer.

## 2 Case presentation

**Figure 1:**
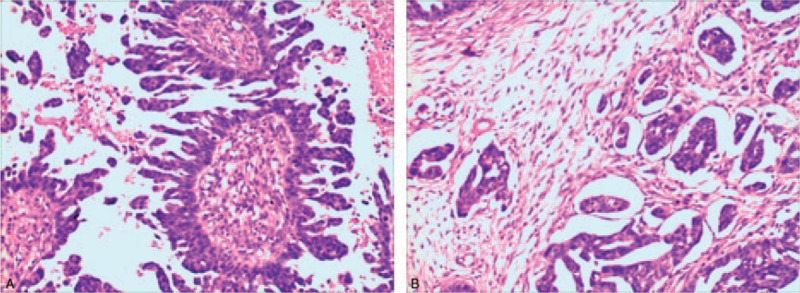
Microscopic view of the tumor (received from pathologist). (A) Ovary serous papillary carcinoma (moderately differentiation), (B) infiltrating adenocarcinoma.

In February 2016, a 51-year-old Chinese woman, diagnosed with ovarian cancer >4.5 years ago at a local hospital, was referred to the Internal Medicine-Oncology department of ShenZhen People's Hospital (ShenZhen, China) due to tumor progression. Postoperative pathological results revealed bilateral ovary serous papillary carcinoma (moderate differentiation) and infiltrating adenocarcinoma (Fig. 1A and B). Cancer cells were found in the uterine body, greater omentum, pelvis, rectum, and anterior abdominal wall. The cancer antigen 125 (CA-125) was 209 μ/mL before the surgery. Then she was operated on January 1, 2012 and administered postoperative chemotherapy. Subsequently, she was treated with 4 types of chemotherapy regimens due to tumor progression or other serious side effects. The 1st 4 types of chemotherapy regimens are listed in Table 1.

**Table 1:**
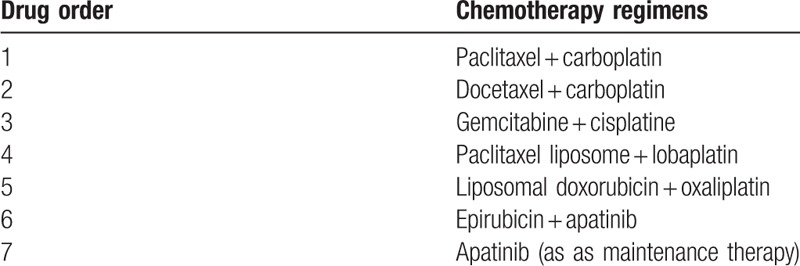
All therapy regimens that the patient has received.

Five months after the last chemotherapy, whole-body positron emission tomography/computed tomography on February 23, 2016 showed subcapsular hepatic and abdominal relapse. The CA-125 level was also significantly increased. The patient then received a new chemotherapy regimen (liposomal doxorubicin combined with oxaliplatin). But the therapy was unsuccessful as the CA-125 remained high. Since the patient was already given several lines of chemotherapy, she could not tolerate the combination of 2 or 3 chemotherapy drugs and drug resistance was almost inevitable. Hence, we started a new regimen of small-molecule VEGFR-2 inhibitor, apatinib (500 mg, qd) combined with chemotherapy of epirubicin (120 mg iv at 4-week intervals) from May 12, 2016. The patient signed the informed consent for administering apatinib as the drug has not been approved for treating ovarian cancer. The patient also provided her written informed consent for the accompanying images to be published in this case study. The case report was approved to be published on Medicine by the Ethical Committee of Shenzhen People's Hospital.

About a week after the initiation of the new regimen, the patient noticed scattered red, point-like rashes in the palms of the hands and soles of the feet, with a tingling sensation. In the 2nd week, the patient progressively developed serious desquamation in the palms and less in the soles of the feet, accompanied by pain and redness. Grade 3 hand–foot syndrome (HFS) was diagnosed (NCI-CTC 3.0). The patient also experienced grade 2 oral ulcer. Therefore, we reduced the dosage of apatinib from 500 to 250 mg/day. Simultaneously, the patient was prescribed oral vitamin B6 and urea cream for oral ulcer and HFS. In the 4th week, the HFS subsided to grade 1. The oral ulcer disappeared.

**Figure 2:**
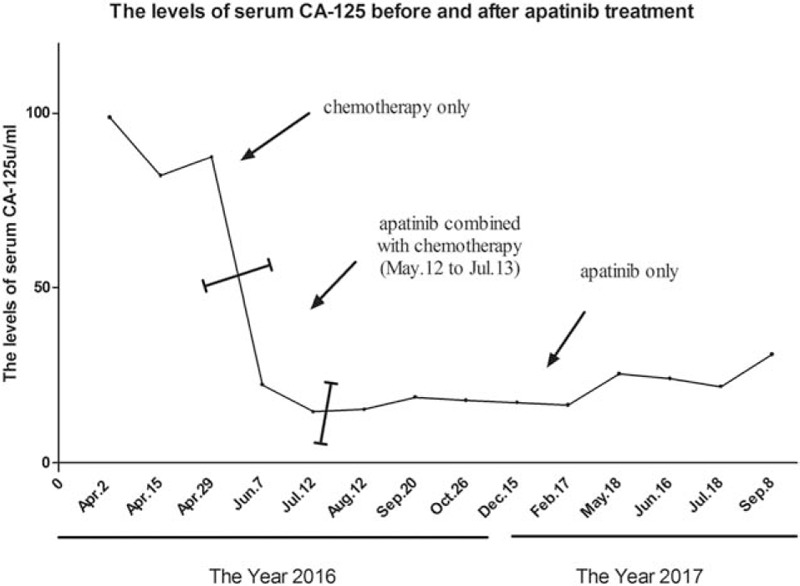
The levels of serum cancer antigen 125 (CA-125) before and after treated by apatinib.

**Figure 3:**
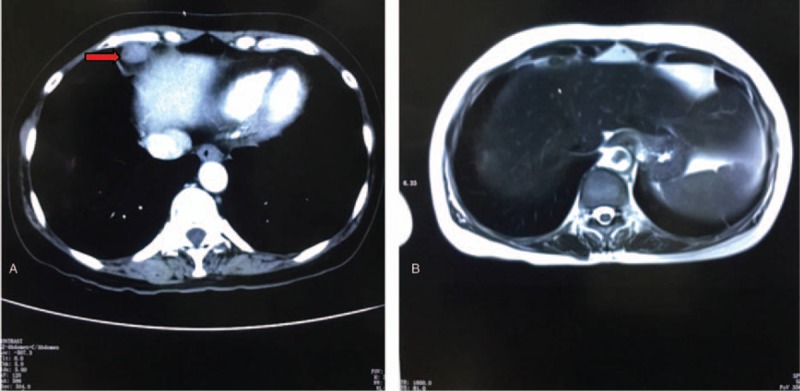
(A) CT image showing liver metastases before and (B) MRI scan showing that the mass on the liver capsule had disappeared after treatment with apatinib and epirubicin. CT = computed tomography, MRI = magnetic resonance imaging.

After 1 cycle of apatinib and epirubicin treatment, the patient's CA-125 quickly reverted to the normal value. The CA-125 values before and after chemotherapy are shown in Fig. 2. Magnetic resonance imaging scan on August 24, 2016 showed that the mass on the liver capsule had disappeared (Fig. 3) as compared to the computed tomography scan on May 9, 2016. The patient continued apatinib as maintenance therapy. By August 12, 2016, the patient suffered mild diarrhea (about 2–3 times/day), but we did not give any treatment as we thought that the symptoms of diarrhea would gradually alleviate or even disappear. Indeed, on September 20, 2016, the patient reported that the symptoms of diarrhea had disappeared several days ago.

After 3 cycles of apatinib and epirubicin treatment from May 12, 2016 to July 13, 2016, the patient continued to use apatinib as maintenance therapy. The patient has been undergoing apatinib treatment for 16 months without major toxic effects except mild HFS and occasional diarrhea (about 2–3 times/day).

## 3 Discussion

As the main type of ovarian cancer, ovarian epithelial carcinoma accounts for 85% to 90% of all ovarian cancers. Moreover, the majority (65%–75%) of women with ovarian cancer are diagnosed with advanced stage disease (III and IV), and only about 15% to 20% of these women are free of disease recurrence at 10 years.^[16]^ Paclitaxel and platinum combination chemotherapy has remained the global 1st-line chemotherapy regimen for patients with ovarian cancer for the past 20 years.^[17]^

Most of these patients easily develop drug resistance during postsurgery or palliative chemotherapy. Therefore, the therapeutic effect is greatly reduced, leading to a survival rate of only 30%. Hence, MDR is the main cause of chemotherapy failure in ovarian cancer.^[2]^

Serum levels of CA-125 are routinely monitored in ovarian cancer patients, and an increase from an individualized nadir concentration is a prognostic indicator of cancer recurrence. The interpretation of CA-125 assay results, with particular focus on ovarian cancer patients undergoing therapy has been extensively reported.^[18,19]^ CA-125 tests and imaging results are routinely used for ovarian cancer patients who are in clinical remission.^[20]^ In this case, the CA-125 quickly reverted to the normal value after treatment with the new regimen. Also, magnetic resonance imaging showed that the original tumor lesions had disappeared as compared to the previous computed tomography images.

Apatinib, a novel oral small-molecule protein TKI, has been demonstrated as a new therapeutic option for various tumor types because of few side effects, convenient usage, and improved outcomes.^[21]^ Nowadays, numerous TKIs are known to be effective as anticancer agents when given alone or in combination with conventional chemotherapeutics.^[22,23]^ Thus, apatinib combined with conventional chemotherapeutics may be a new option to inhibit the growth of ovarian cancer, especially for patients who have previously received several lines of chemotherapy or developed drug resistance to chemotherapy. Apatinib can inhibit tumor angiogenesis and reverse MDR by inhibiting their transport function.^[4,24]^ However, no case has been reported for apatinib in the treatment of ovarian cancer.

Although the mechanisms leading to MDR are not fully established, increased drug efflux via overexpression and increased activity of MDR pumps, such as P-glycoprotein (P-gp) are well known.^[25,26]^ Resistance to anticancer drugs remains a major obstacle to successful chemotherapy. Most cancer deaths, if not all, are caused by chemotherapy failure because tumors quickly develop drug resistance.^[27]^ The patient in this case had prolonged exposure to paclilaxel, platinum, gemcitabine, and adriamycins. Cancer cells are known to simultaneously acquire resistance not only to the specific drug used but also a broad spectrum of drugs that are structurally and functionally unrelated to each other.

Apatinib was demonstrated to reverse ABCB1/P-gp/MDR1 and ABCG2/BCRP-mediated MDR by inhibiting their transport function, but not by blocking the AKT or ERK1/2 pathways or by downregulating the expression of ABCB1/P-gp/MDR1 or ABCG2/BCRP.^[4,24]^ The antitumor activity of apatinib has been studied in vitro and in vivo.^[8]^ In vitro, apatinib potently suppressed the kinase activities of VEGFR-2, c-Kit, and c-Src, and inhibited cellular phosphorylation of VEGFR-2, c-Kit, and PDGFR-β.^[8,28]^ Apatinib also showed antitumor efficacy in vivo when administrated alone or in combination with chemotherapy against various established tumor xenografts with good tolerance.^[8,29,30]^ In fact, more than 90% of patients with malignant tumors die of MDR.^[8,31]^ Apatinib could also reverse cancer MDR mediated by MDR protein 1 (ABCB1), MDR-associated protein 1 (MARP1), and breast cancer resistant protein (BCRP) through inhibiting their transport functions.^[5]^ Thus, apatinib may be useful in overcoming MDR to other conventional antineoplastic drugs.

In addition to the well-known effects of VEGF in angiogenesis, recent data suggest that autocrine VEGF signaling in tumor cells plays an important role in promoting their proliferation and inhibiting apoptosis.^[32]^ After a certain tumor size is reached, the existing blood vessels become insufficient and new blood vessels are required to continue tumor growth. Angiogenic phenotype can result from genetic or local environmental changes that lead to the activation of endothelial cells.^[33]^ Blocking this process may prevent growth of small tumor deposits and improve survival of patients. VEGF is one of the key elements released by cancer cells for the stimulation of angiogenesis and binds to a receptor on endothelial cells (VEGF-R).^[34]^ Another advantage is that tumor endothelial cells are not malignant, and unlike cancer cells, they do not have preexisting mutations that favor the development of further mutations, which could lead to drug resistance. In addition, antiangiogenic agents may work synergistically with conventional chemotherapeutic agents or other novel systemic agents, due to their different mechanisms of action.

Apatinib is a multitargeted TKI which could selectively inhibit VEGFR-2 and also mildly inhibit c-Kit and c-Src tyrosine kinases.^[7]^ Thus, the adverse effects (AEs) of apatinib were various. In our case, the patient has experienced several AEs including oral ulcer, HFS, and diarrhea. These AEs are common according the previous clinical trials, case reports, and our clinical experience.^[35]^ All this AEs were well controlled after appropriate treatment. Apatinib is an orally small-molecule VEGFR-2 inhibitor which could disrupt endothelial and vascular repair mechanisms, resulting in persistent damage to vessels and fibroblasts at areas of frequent trauma or friction (such as the palms of the hands, soles of the feet, and elbows). Vascular competence is important for tissue repair, and thus vascular damage can impair the skin's ability to recover from day-to-day wear and tear.^[36]^ This may explain the occurrence of HFS. Although we did not know exactly the mechanism of diarrhea and oral ulcer, these 2 AEs were not too serious to affect the treatment compliance of our patient and could be controlled well by empirical therapy.

The standard regime for ovarian cancer is chemotherapy with 2 drugs. In our patient, paclitaxel, platinum, gemcitabine, and adriamycins (Table 1) could not control tumor progression. The combination of 2 chemotherapeutic agents often causes numerous side effects, especially in patients receiving long-term systemic chemotherapy. Our patient had late stage ovarian cancer when diagnosed and received 5 combined chemotherapy regimens in almost 5 years before apatinib was used. However, the combination of 2 chemotherapy drugs could not control the tumor. On the contrary, it would probably increase the drug toxicity. Besides, the cancer cells develop drug resistance over time. Since our patient had already received several lines of chemotherapy and had developed drug resistance, we used apatinib combined with chemotherapy of epirubicin and apatinib monotherapy as maintenance therapy to successfully control the cancer with a complete response.

## 4 Conclusions

In conclusion, MDR is the main cause of chemotherapy failure in ovarian cancer. The successful treatment of our patient shows that apatinib combined with chemotherapy may provide an additional option for the treatment of ovarian cancer, especially adenocarcinomas. Apatinib could also be used as maintenance therapy after ovarian cancer is controlled. The success achieved in our case may be due to apatinib could reverse MDR and inhibits tumor angiogenesis. Further studies are needed to optimize the therapeutic regimen.

## Acknowledgements

The authors thank the patient for his participation and his agreement to publication of the report.
